# Radiotherapy combined with PD‐1/PD‐L1 inhibitors in NSCLC brain metastases treatment: The mechanisms, advances, opportunities, and challenges

**DOI:** 10.1002/cam4.5016

**Published:** 2022-08-19

**Authors:** Zi‐Ying Chen, Xiao‐Tong Duan, Si‐Miao Qiao, Xiao‐Xia Zhu

**Affiliations:** ^1^ Department of Radiation Oncology, Zhujiang Hospital Southern Medical University Guangzhou China

**Keywords:** brain metastases, immunotherapy, mechanism, non‐small‐cell lung cancer, radiotherapy, safety, timing

## Abstract

At present, whole‐brain radiation therapy/stereotactic radiosurgery is one of the main local treatments for brain metastasis of non‐small‐cell lung cancer (NSCLC). Currently, it has been proved that radiotherapy (RT) can regulate the immune response, and small‐sample studies have shown that patients with NSCLC brain metastases (BMs) can benefit from RT combined with immunotherapy (IO). However, the efficacy and safety of the combination treatment have not been deeply elaborated. Notably, as a challenge that is still being explored, the timing of RT combined with IO is likely to be an important factor affecting efficacy and prognosis. This article reviews the current application and challenges of RT combined with IO from the perspectives of molecular mechanism, combination timing, safety, and efficacy. The purpose is to provide information on clinical evidence‐based medicine of combination between RT with IO. For further investigation, we also discuss the major challenges and prospects of RT combined with IO in NSCLC BMs.

## Graphical abstract

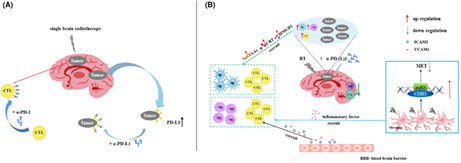

## 1 INTRODUCTION

Lung cancer is the leading cause of cancer‐related deaths in the world. Non‐small‐cell lung cancer (NSCLC) accounts for about 85% of lung cancers, and more than half of the patients are advanced at the first diagnosis.^1^ The incidence rate of brain metastases (BMs) has been reported at 20%–56% during the course of the disease.^2^ Compared with the limited role of conventional cytotoxic chemotherapy or early targeted therapy in controlling BMs due to its incapability to crossing the blood–brain barrier (BBB), local treatments including whole‐brain radiotherapy (WBRT) and stereotactic radiosurgery (SRS) have been widely utilized for BMs in NSCLC.

At present, programmed death‐1/programmed death ligand‐1 (PD‐1/PD‐L1) inhibitors, including pembrolizumab, atezolizumab, nivolumab, durvalumab, etc, exhibited encouraging results in clinical trials. Pembrolizumab has been recommended as a first‐line treatment for advanced NSCLC patients with high PD‐L1 expression. Despite these achievements, only a part of patients benefited from PD‐1/PD‐L1 inhibitors. Additionally, PD‐1/PD‐L1 inhibitors resistance might eventually develop. To improve the prognosis and overcome resistance, some clinical studies have also suggested that PD‐1 and PD‐L1 inhibitors combined with radiotherapy (RT) may be expected to improve the anti‐cancer efficacy.^3^, ^4^, ^5^ Preclinical studies have shown that RT can regulate the immune system, including inducing the abscopal effect, increasing the infiltration and activity of tumor‐related lymphocytes, and elevating the number of tumor neoantigens.^6^ The combination of PD‐1/PD‐L1 inhibitors with WBRT/SRS should be evaluated in the era of immunotherapy (IO). In this review, we will discuss the mechanisms, advances, opportunities, and challenges of the combination of RT and PD‐1/PD‐L1 inhibitors for BM in patients with NSCLC.

## 2 THE POTENTIAL MECHANISMS OF RT AND PD‐1/PD‐L1 INHIBITORS IN ANTI‐TUMOR IMMUNITY

**FIGURE 1:**
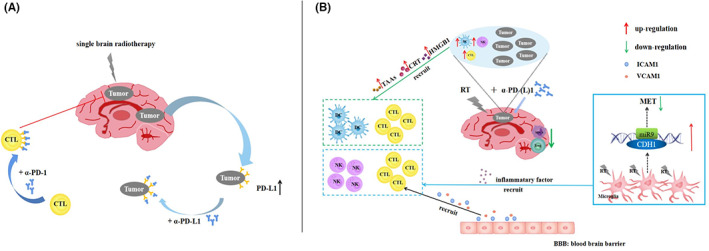
The theoretical basis of radiotherapy combined with immunotherapy. (A) PD‐1 inhibitors can reverse the immunosuppressive microenvironment induced by radiotherapy. (B) Radiotherapy combined with immunotherapy sequentially or concurrently. ɑ‐PD‐1, ɑ‐programmed death‐1; BBB, blood–brain barrier; CDH1, cadherin 1; CRT, calreticulin; CTL, cytotoxic T lymphocyte; DC, dendritic cell; HMGB1, high mobility group box‐1 protein; ICAM‐1, intercellular cell adhesion molecule‐1; MDSCs, myeloid‐derived suppressor cells; MET, mesenchymal‐epithelial transition; miR9, microRNA 9; NK, natural killer cell; PD‐L1, programmed death‐ligand 1; RT, radiotherapy; SBS, stereotactic radiosurgery; TAAs, tumor‐associated antigens; Tregs, regulatory T cells; VCAM‐1, vascular cell adhesion molecule‐1; WBRT, whole‐brain radiotherapy.

The role of RT in immunomodulation has gained extensive attention. WBRT/SRS could cause immunogenic cell death, promote the release of tumor‐associated antigens, increase the number of cytotoxic T lymphocytes (CTLs)^7^, ^8^ to improve the immunocompetence of patients. The immunogenic cell death triggers the extracellular release of high mobility group box‐1 protein and transfer of calreticulin to the cell surface, which activates CTLs, dendritic cells, and other immune cells.^9^, ^10^, ^11^ Moreover, RT can also increase the permeability of the BBB. In addition, RT activates microglia and induces microglia polarization to M1 type accompany by secreting miR‐9 to regulate mesenchymal‐to‐epithelial transition (MET) of tumor cells through Cadherin 1 and reduce the colonization of BMs.^12^ It is observed that M1‐type microglia also recruit various immune cells by secreting pro‐inflammatory factors, such as natural killer cells and CTLs.^13^ Radiation also increases the MHC class I receptors on various antigen‐presenting cells and enhances the reactivity of T‐cell immune response.^14^ Thus, WBRT or SRS provides a more active immune microenvironment for PD‐1/PD‐L1 inhibitor treatment.^8^ It is generally acknowledged that PD‐L1 expression was an important prognostic indicator of NSCLC patients.^15^ The increased PD‐L1 expression induced by radiation could make patients more sensitive to PD‐1/PD‐L1 inhibitors, which gain a better response rate and extends overall survival (OS).^16^, ^17^, ^18^ Current evidence suggests that the combination of WBRT with αPD‐1 is involved in a variety of immunomodulatory processes and plays a significant role in anti‐tumor immunity.^19^ The combination of WBRT or SRS and PD‐1/PD‐L1 inhibitors reduces myeloid‐derived suppressor cells and regulatory T cells (Tregs) to inhibit the immunosuppressive microenvironment^20^, ^21^ and induce the expression of vascular cell adhesion molecule‐1 and intercellular cell adhesion molecule‐1 on the endothelial cells of BBB.^22^, ^23^ These adhesion molecules are associated with T‐cell recruitment.^24^ In conclusion, RT combined with immune checkpoint inhibitors (ICIs) plays a collaborative role in the treatment of BMs by affecting the tumor microenvironment and improving immune function within the brain (Figure 1). Summarily, based on the immunomodulatory effect and abscopal response of RT, the combination of RT with IO appears to be one of the most promising treatment protocols for NSCLC.

## 3 ADVANCES IN SYNERGISTIC EFFECTS OF RT AND PD‐1/PD‐L1 INHIBITORS FOR NSCLC BMS TREATMENT

RT and IO have synergistic effects in clinical (Table 1). In a comparative analysis of survival with intracranial RT plus IO versus no IO in 13,998 patients with BMs from the US National Cancer Database, the median OS with ICIs was 13.1 months (13.1 vs. 9.7 months) and the 3‐year OS rate was 17% (17% vs. 12%), higher than that of non‐recipients. Paired multivariate comparisons showed that ICIs are an independent predictor of increased OS in NSCLC patients with intracranial metastases.^25^ In addition, the single‐center secondary analysis of the KEYNOTE‐001 showed that median progression‐free survival (PFS) was longer in patients who received RT (cerebral or extracranial) in combination with pembrolizumab than in patients who received pembrolizumab alone.^26^ Furthermore, a retrospective analysis of SRS combined with nivolumab or durvalumab revealed that the Kaplan–Meier rates of OS at 6 and 12 months were 48/41% from the date of SRS, and 81/51% from the date of cranial metastases diagnosis, respectively.^27^ Similarly, Chen et al. reported that the median survival time was 12.9 months (RT alone), 14.5 months (non‐concurrent), and 24.7 months (concurrent) from 260 patients (157 NSCLC) receiving SRS with/without ICI.^28^ Additionally, a retrospective multicenter study showed that prolonged median survival was 192 days for patients with NSCLC BMs who were treated by WBRT/SRS combined with PD‐1 inhibitors, compared with historical controls.^29^ In summary, these results suggested that NSCLC patients benefited from the combination of RT and IO, ongoing trials will further help us obtain a comprehensive understanding of the combination treatment, thus guiding clinical practice to achieve superior survival benefits.

**TABLE 1:** Advances in synergistic effects of radiotherapy and PD‐1/PD‐L1 inhibitors for NSCLC BMs treatment

Author (year)	Number of cases	Means of intervention	Radiotherapy plan	Immunotherapy plan	Outcome
Patruni et al (2019)^25^	13,998	RT + IO (545) vs. RT (13,545)	/	/	Median OS:13.1 vs. 9.7 months 3‐year OS: 17% vs. 12%
Shaverdian et al (2017)^26^	97	Extracranial RT + IO (38) vs. IO (59)	/	Pembrolizumab (2 mg/kg or 10 mg/kg, q3w, iv; or 10 mg/kg, q2w, po)	Median PFS: 6.3 months vs. 2.0 months; 6‐month PFS: 54% vs. 21%
Ahmed et al (2017)^27^	17	RT + IO	SRS or FSRT, 18–24 Gy/F or 25 Gy/5F	Nivolumab or Durvalumab	OS KM rates (6/12 months): 48%/81% (from the date of SRS); 81%/51% (from the date of cranial metastases diagnosis)
Chen et al (2018)^28^	260 (157 NSCLC)	SRS/SRT (181) vs. non‐concurrent SRS/SRT + IO (51) vs. concurrent SRS/SRT + IO (28)	SRS/SRT, 15–24 Gy/1F, 18–24 Gy/3F or 25 Gy/5F	Ipilimumab, Nivolumab, or Pembrolizumab	Median OS: 12.9 months (SRS/SRT) vs. 14.5 months (non‐concurrent SRS/SRT + IO) vs. 24.7 months (concurrent SRS/SRT + IO)
Pike et al (2017)^29^	85 (39 NSCLC)	SRS/WBRT + IO	WBRT (12–39 Gy)/SRS (15–30 Gy)	Pembrolizumab, Nivolumab or both (3 mg/kg)	Median OS: 192 days

Abbreviations: BMs, brain metastases; FSRT, fractionated stereotactic radiotherapy; IO, immunotherapy; iv, intravenous injection; KM, Kaplan–Meier; NSCLC, non‐small‐cell lung cancer; OS, overall survival; PD‐1/PD‐L1, programmed death‐1/programmed death ligand‐1; PFS, progression‐free survival; po, per os; q2w, 2 weeks using a; q3w, 3 weeks using a; RT, radiotherapy; SRS, stereotactic radiosurgery; SRT, stereotactic radiotherapy; WBRT, whole‐brain radiothera.

## 4 THE OPTIMAL TIMING FOR RT COMBINED WITH ICIS

Currently, the optimal timing for combining RT and PD‐1/PD‐L1 inhibitors remains to be elucidated and might have a significant effect on the generation of a potent and durable anti‐tumor effect. The conclusions of the researches conducted by various research cohorts are different (Table 2). Li et al. demonstrated that the median time of intracranial PFS was 9.7 months, and the intracranial PFS rate was 75% at 4 months by concurrent treatment of SRS combined with nivolumab/ipilimumab.^30^ Moreover, better OS (1‐year OS: 70.2%) and Regional control (1‐year local progression‐free interval: 78.9%) were demonstrated in the “concurrent SRT and IO” group.^31^ Nonetheless, some studies have been performed to evaluate concurrent or sequential (nivolumab or pembrolizumab) and RT in patients with stage IV NSCLC. PFS and median OS for patients who received RT prior to, concurrently, or post‐IO were 7.8/17.5, 9.2/23.4, and 5.7/14.4 months, respectively. The data showed that PFS and OS in the concurrent arm were better than the sequential arm.^32^ Similarly, a retrospective study conducted by Chen et al. (2015) evaluate the efficacy and safety of ipilimumab/nivolumab plus SRS for patients with intracranial diseases. The results showed that concurrent SRS with ipilimumab/nivolumab could reduce the incidence of new intracranial diseases and tumor burden.^33^ Another retrospective study performed by Srivastava and Huang showed that concurrent RT with ICIs significantly improved the 6‐month local control rate (76% vs. 100%) and 6‐month brain control rate (41% vs. 71%) in patients with NSCLC BMs, compared to the non‐concurrent group.^34^ Related to this, a preclinical study showed that compared to receiving sequential ICIs and RT, concurrent RT and ICIs had a better anti‐tumor effect.^35^

**TABLE 2:** The optimal timing for radiotherapy combined with ICIs

Author (year)	Number of cases	Intervention time	Radiotherapy plan	Immunotherapy plan	Outcome
Li et al (2020)^30^	13	Concurrent RT + IO (SRS within 7 days of IO)	SRS	(Nivolumab, 3 mg/kg, q2w + Ipilimumab, 1 mg/kg, q6w) × 4 cycles + Nivolumab, 450 mg, q4w.	Intracranial mPFS: 9.7 months; 4‐month PFS rate: 75% Extracranial ORR: 33%
Porte et al (2021)^31^	51	“SRT before IO” vs. “concurrent SRT + IO” (IO within 1 month of SRT) vs. “SRT after IO”	SRT (15–21 Gy/F, 56.0% or 18–27 Gy/3F, 41.8%)	Nivolumab (47.1%), Pembrolizumab (33.3%), Durvalumab (15.7%), or Atezolizumab (3.9%) (for a median duration of 4.9 months)	1 year R‐PFI: 24.1% vs. 49.6% vs. 34.2%; 1 year OS: 67.5% vs. 70.2% vs. 69.2%; 1‐year L‐PFI: 70.1% vs. 78.9% vs. 77.8%
Srivastava et al (2017)^34^	50 (24 NSCLC)	RT + adjuvant IO (applying PD‐1 inhibitors more than 3 weeks after SRS) (23) vs. Concurrent RT + IO (applying PD‐1 inhibitors at or <3 weeks before SRS) (27)	SRS	Nivolumab/Pembrolizumab	6‐month LC (76% vs. 100%) 6‐month DBC (41% vs. 71%)
Imber et al (2017)^36^	45	Sequential IO + brain RT (RT >2 months after last IO) (36%) vs. Concurrent brain RT + IO (64%)	SRS (2100 cGy)/hRT (3000 cGy/5F)	Anti PD‐(L)1	Median DBF:4.9 months vs. 3.9 months

Abbreviations: DBC, distant brain control; DBF, distant brain control; hRT, hypofractionated radiotherapy; ICIs, immune checkpoint inhibitors; IO, immunotherapy; LC, local control; L‐PFI, local progression‐free interval; mPFS, median progression‐free survival; NSCLC, non‐small‐cell lung cancer; ORR, objective response rate; OS, overall survival; PD‐1, programmed death‐1; PD‐L1, programmed death‐ligand 1; PFS, progression‐free survival; q2w, 2 weeks using a; q4w, 4 weeks using a; R‐PFI, regional progression‐free interval; RT, radiotherapy; SRS, stereotactic radiosurgery; SRT, stereotactic radiotherapy.

For the optimal timing of the combination, there is no certain consensus on whether PD‐1/PD‐L1 inhibitors should be administrated concurrent, before or after the initiation of RT. Imber et al. observed the median time of distant brain failure (defined as the presence of a new brain metastatic tumor) in patients with NSCLC BMs who received PD‐(L)1 based IO sequentially or concurrently combined with SRS/hypofractionated radiation therapy were 4.9 and 3.9 months, respectively.^36^ Additionally, Shepard et al. carried out a retrospective paired cohort study regarding combined ICIs (nivolumab, pembrolizumab, or atezolizumab) with SRS, which proved that compared to the ICI‐naive group, the BMs shrinkage time was shorter without increasing the incidence of peritumoral edema or radiation necrosis in the concurrent group. However, the comparison of OS and PFS between the two groups was not statistically different.^37^ Moreover, a survey of 462 European doctors showed that 49.4% thought ICIs should not be used during WBRT, while 44.6% thought ICIs should not be used during SRS.^38^ The RTOG 3505 study showed that palliative RT required at least a 14‐day window period before using nivolumab.^39^ Although the addition of RT may enhance the immune response, the combination of IO with RT requires more effort to optimize the dose and schedule.^40^ Based on the current data, RT concomitant with PD‐(L)1‐based IO has shown considerable survival benefits, but a few researchers have argued otherwise. Therefore, prospective head‐to‐head clinical trials are warranted to provide more evidence and further guide clinical research and practice.

## 5 SAFETY AND TOLERANCE OF RT COMBINED WITH ICIS

Most small samples and retrospective clinical studies have shown that RT combined with ICIs was safe (Table 3). All toxic side reactions were below Grade 4 during treatment of SRS combined nivolumab, atezolizumab, or pembrolizumab on NSCLC BMs.^41^ A retrospective study conducted by Hubbeling et al. showed that the rate of adverse events (AEs) at all grades in the ICIs treatment group and the ICIs‐naive treatment group was comparable to any type of in patients with NSCLC BMs. The most frequently observed in the Grade 3 (G3) or worse AEs of ICIs‐treated queue were headaches, anorexia, and cognitive impairment.^4^ Li et al. reported that except for only one patient with a dose limit‐related toxic reaction, three patients (25%) developed treatment‐related G3 events, and one patient developed G4 events in the Phase I/II single‐center trial about concurrent SRS combined nivolumab/ipilimumab.^30^ The most common AE we concerned about is treatment‐related neurotoxicity. Although there was a strong correlation between symptomatic radioactive necrosis (RN) and ICIs in patients with NSCLC treated with SRS‐SRT and newly diagnosed BMs,^42^ the AEs are less obvious. Many studies have shown that RT combined with ICIs does not increase the risk of neurotoxicity,^28^, ^43^ and even this combination rarely presents neurotoxicity^27^, ^44^ that requires intervention with drugs.

**TABLE 3:** Safety and tolerance of RT combined with ICIs

Author (year)	Number of cases	Means of intervention	Radiotherapy plan	Immunotherapy plan	Outcome (AEs)
Li et al (2020)^30^	13	Concurrent RT + IO	SRS	(Nivolumab, 3 mg/kg, q2w + Ipilimumab, 1 mg/kg, q6w) × 4 cycles + Nivolumab, 450 mg, q4w	G3 seizures (1) G3 events (increased liver function, fatigue, nausea, adrenal insufficiency and myocarditis) (3) G4 events (pneumonitis/acute respiratory distress syndrome) (1)
Matteucci et al. (2019)^44^	12 (10 NSCLC)	RT + IO	SRS/WBRT	Anti PD‐1	RN (1) Seizures (1)
Imber et al (2017)^36^	45	Sequential IO + brain RT (RT >2 months after last IO) (36%) vs. Concurrent brain RT + IO (64%)	SRS (2100 cGy)/hRT (3000 cGy/5F)	Anti PD‐(L)1	RN (3 vs. 2)
Ahmed et al (2017)^27^	17	RT + IO	SRS or FSRT, 18–24 Gy/F or 25 Gy/5F	Nivolumab or durvalumab	Colitis (1) Pneumonia (1)
Schapira et al (2018)^41^	37	RT + IO	SRS 15–25 Gy/1–5F	Nivolumab (83.8%), atezolizumab (10.8%), pembrolizumab (5.4%)	Neurotoxicity<grade 4 (4) RN (3)
Arscott et al (2019)^45^	78 (24% NSCLC)	WBRT + ICIs (21) vs. SRS + ICIs (57)	SRS/WBRT (20 Gy/5F, 30 Gy/10F or 37.5 Gy/15F)	Pembrolizumab (36%), Ipilimumab (32%), Nivolumab (24%)	Neurotoxicity (≥ grade 2) (WBRT, 38% vs. SRS, 23%)
Kotecha et al (2019)^47^	150 (99 NSCLC)	RT + IO	SRS/WBRT	Nivolumab/Pembrolizumab/Atezolizumab/Avelumab	RN (3.2%)
Hubbeling et al (2018)^4^	163	CRT + ICIs (50) vs. CRT (113)	SRS/WBRT/PBI	Anti PD‐(L)1	ICIs‐treated: headaches (*n* = 2), anorexia (*n* = 2), cognitive impairment (*n* = 2) ICIs‐naive: fatigue (*n* = 4), movement disorder (*n* = 4), epilepsy (*n* = 3), pathologically confirmed RN (*n* = 2), grade 4 evevt (central nervous system necrosis) (1)
Chen et al (2017)^28^	260 (157 NSCLC)	SRS/SRT (181) vs. non‐concurrent SRS/SRT + IO (51) vs. concurrent SRS/SRT + IO (28)	SRS/SRT, 15–24 Gy/1F, 18–24 Gy/3F or 25 Gy/5F	Ipilimumab, Nivolumab, or Pembrolizumab	Total pathological RN = 3%
Singh et al (2019)^43^	85	IO + RT (39) vs. RT + chemotherapy (46)	SRS	Nivolumab (20), Pembrolizumab (14), Ipilimumab/Nnivolumab (4), Atezolimumab (1)	Incidence rate of RN: 10.2% (4/39) vs. 10% (5/46) Occurrence time of RN: 11.7 months vs. 29.6 months

Abbreviations: CRT, chemoradiotherapy; hRT, hypofractionated radiotherapy; ICIs, immune checkpoint inhibitors; IO, immunotherapy; NSCLC, non‐small‐cell lung cancer; PBI, partial brain irradiation; PD‐1, Programmed death‐1; PD‐L1, programmed death‐ligand 1; q2w, 2 weeks using a; q4w, 4 weeks using a; RN, radionecrosis; RT, radiotherapy; SRS, stereotactic radiosurgery; SRT, stereotactic radiotherapy; WBRT, whole‐brain radiation therapy.

The timing of RT combined with ICIs may not be a factor for the increase in toxicity.^45^ However, the study had not yet reported the results of the evaluation of cognitive function.^44^ Imber et al. (2017) found no acute Grade 3–4 AEs or new intracranial bleeding caused by brain RT in neither concurrent group nor sequential group. This study showed that IO combined with high‐dose brain RT was safe as well.^36^, ^40^ A multi‐center retrospective study showed no correlation between an increased risk of severe or accidental toxic side effects in a combination of and nivolumab treatment for NSCLC patients with metastasis.^46^ Kotecha et al. found that the timing of RT combined with ICI did not affect the risk of toxicity. In patients treated with chemo‐radiotherapy (CRT) and immediate (±1 half‐life) ICIs, none required surgery even though some of them had symptomatic RN.^47^ In conclusion, the occurrence of AEs would not increase between the different treatment sequences of ICIs and CRT. Longer and rigorous follow‐up data need to be obtained for a comprehensive understanding of the toxicities from the combination treatment.

## 6 DISTINGUISH PSEUDO AND TRUE PROGRESSION DURING RT COMBINED WITH ICIS

For patients receiving ICIs and RT, the pseudo‐progression (Psp) of BMs should be taken into consideration. Psp meant that the tumor lesions initially expanded and then the tumor improves spontaneously. It is a common phenomenon that may be necrosis or inflammatory cell infiltration after IO. Biopsy, imaging follow‐up, and evaluation of patient's functional status are standard procedures to distinguish between pseudo and true progression.^48^ Akhoundova et al. (2018) observed Psp in 4/27 patients of NSCLC BMs who received nivolumab or pembrolizumab combined with WBRT/SRS by PET examination. Similarly, Akhoundova et al. (2019) reviewed 53 patients with NSCLC BMs who received ICIs and RT and then received ^18^F‐FET PET scans after the progression of BMs, and the results showed that ^18^F‐FET PET correctly identified Psp in nine of the 11 patients (81.8%). ^18^F‐FET PET might be helpful to distinguish between pseudo and true progression in the RT and ICIs combination treatment.^49^

## 7 CONCLUSION AND FORESIGHT

Currently, RT combined with IO becomes one of the most important trends in the treatments of NSCLC BMs. Furthermore, lots of clinicians support this combination treatment in oligometastases or advanced stage in NSCLC BMs, but the evidence‐based researches and clinical data about this combination are scarce. In this review, we concluded that the mechanism of RT combined with ICIs mainly focused on affecting immunogenic cell death,^9^ MET,^12^ BBB permeability,^22^, ^23^ and activating anti‐tumor responses by improving immunocompetence.^7^, ^24^ It indicates that RT combined with IO has a synergistic effect, but the deeper mechanism and the relationship among the current mechanisms need to be further explored. At present, most studies are still in the preclinical stage, more research is needed to prove the molecular biological evidence of RT combined with IO.

Nowadays, the main combination regimen in NSCLC BMs is the combination of brain RT and ICIs. In terms of the data in our review, concurrent RT combined with ICIs has a relatively high control rate of intracranial progression^33^ and a better prognosis.^32^, ^37^ However, some studies support the practice of RT before IO.^39^, ^40^ Both concurrent and sequential RT combined with IO have different efficacy, but most studies have advocated that RT concurrently combined with PD‐(L)1‐based IO has a better survival benefit in NSCLC BMs, compared with the sequential mode. Interestingly, according to the researches included in our paper, the mode of RT and timing of RT combined with IO may not be factors for adverse reactions. Due to the short follow‐up period, there is a limitation of not observing the time of late adverse reactions. Therefore, to determine a more beneficial combined RT regimen and the timing of the combination of IO and RT, more head‐to‐head, prospective, and multi‐center clinical researches are needed to be carried out. At the same time, future studies need to extend the follow‐up time to avoid missing late AEs.

In the exploration of the anti‐tumor efficacy of RT combined with ICIs, it is inevitable to encounter the situation of Psp. Currently, the reliability of identifying progression is usually increased by biopsy, clinical status, imaging follow‐up, and other means.^48^ However, there is no clear standard for the diagnosis of Psp in clinical practice. Therefore, it is expected that relatively accurate and non‐invasive technologies can be developed or relevant statistics of large data researches can help clinical diagnosis of the true progression of tumors.

There are some limitations in this review. The first part of the results of RT combined with IO is the OS data of BMs in a variety of tumors, although including NSCLC, these studies did not independently explore the survival data of BMs in NSCLC. Secondly, some studies included WBRT and SRS as the same intervention to collect the outcome data, so that they could not distinguish the differences and advantages of WBRT and SRS combined with IO, respectively. In addition, pretreatment in some studies is not described, which may ignore the effect of pretreatment on results. Finally, some of these studies are only in abstract form, which limits understanding of the specific design scheme of these studies, and descriptions of the results are also in abstract form. However, the overall direction is supportive of RT combined with IO for BMs in NSCLC.

Most of the current clinical evidence on the safety and importance of ICIs combined with RT to NSCLC BMs is retrospective. For patients with NSCLC BMs, the efficacy of other ICIs remain unknown, whose targets are T‐cell immunoglobulin‐3 (Tim‐3), OX40 (CD134), 4‐1BB (CD137), pyridine 2, 3‐dioxygenase‐1, killer cell immunoglobulin‐like receptors. To date, clinical studies on RT combined with PD‐1/L1 inhibitors for NSCLC BMs are ongoing (Table 4), such as NCT04889066, NCT04787185, NCT04650490, etc. Therefore, further translational medicine research and large‐scale prospective studies are necessary to better reveal the interactions and combinations of RT and IO to optimize treatment options for the patients with NSCLC BMs.

**TABLE 4:** Summary of ongoing clinical trails of RT combined with IO for BMs in NSCLC

NCT number	Title	Conditions	Interventions	Status	Study type	Phase	Locations
NCT05021328	Toripalimab combined with anlotinib and SBRT in patients with untreated brain metastases of driven gene negative NSCLC	BMs from NSCLC	Drug: Toripalimab Drug: Anlotinib Radiation: SBRT	Recruiting	Interventional	Phase 1	Hubei Cancer Hospital, Wuhan, Hubei, China
NCT04889066	Durvalumab (MEDI4736) and Radiosurgery (FSRT vs. PULSAR) for the treatment of non‐small‐cell lung cancer brain metastases	BMs from NSCLC	Drug: Durvalumab Radiation: SRT	Not yet recruiting	Interventional	Phase 2	University of Texas, Southwestern Medical Center, United States
NCT04787185	Stereotactic radiotherapy in association with immunotherapy for the treatment of NSCLC brain metastases	BMs from NSCLC	Drug: IO Radiation: RS/HFSRT	Recruiting	Observational	/	Radioterapia Oncologica AOU Careggi, Firenze, FI, Italy
NCT04768075	Camrelizumab combined with SRT/WBRT and Chemotherapy in patients with brain metastases of driven gene negative NSCLC	BMs from NSCLC	Drug: Camrelizumab Drug: Placebo Drug: Cisplatin Drug: Carboplatin Drug: Pemetrexed Drug: Paclitaxel Drug: Albumin paclitaxel Radiation: SRT/WBRT	Not yet recruiting	Interventional	Phase 3	Guangdong Association of Clinical Trials, China
NCT04650490	SRS timing with immune checkpoint inhibition in patients with untreated brain metastases from non‐small‐cell lung cancer	BMs from NSCLC	Drug: IO Radiation: SRS	Recruiting	Interventional	Phase 2	Duke Cancer Center, Durham, North Carolina, United States
NCT04345146	Bevacizumab combined with fractionated stereotactic radiotherapy for 1–10 BMs from non squamous NSCLC	BMs from non squamous NSCLC	Drug: Bevacizumab Radiation: FSRT	Unknown status	Interventional	Phase 2	Sun Yat‐sen University, Guangzhou, China
NCT04291092	Camrelizumab combined with local treatment in NSCLC patients with BM	BMs From NSCLC	Drug: Camrelizumab Drug: Chemotherapy Radiation: WBRT	Recruiting	Interventional	Phase 2	Zhejiang Cancer Hospital, Hangzhou, Zhejiang, China
NCT04180501	SRS sequential Sindilimab in brain metastasis of NSLSC	BMs from NSCLC	Drug: Sintilimab Radiation: SRS	Unknown status	Interventional	Phase 2	Union hospital, Wuhan, Hubei, China
NCT02978404	Combining radiosurgery and Nivolumab in the treatment of brain metastases	BMs from clear‐cell metastatic renal cell carcinoma/NSCLC/small cell lung cancer/melanoma	Drug: Nivolumab Radiation: RS	Active, not recruiting	Interventional	Phase 2	Centre Hospitalier de l'Université de Montréal, Montreal, Quebec, Canada
NCT02858869	Pembrolizumab and Stereotactic radiosurgery for melanoma or non‐small‐cell lung cancer brain metastases	BMs from melanoma/NSCLC	Drug: Pembrolizumab Radiation: SRS	Active, not recruiting	Interventional	Phase 1	Emory University/Winship Cancer Institute, Atlanta, Georgia, United States
NCT02696993	Nivolumab and radiation therapy with or without Ipilimumab in treating patients with brain metastases from non‐small‐cell lung cancer	BMs from NSCLC	Drug: Ipilimumab Drug: Nivolumab Radiation: SRS/WBRT	Recruiting	Interventional	Phase 1 Phase 2	M D Anderson wCancer Center, Houston, Texas, United States
NCT01891708	VEGFRs predict bevacizumab benefit in advanced non‐small‐cell lung cancer	BMs from NSCLC	Drug: bevacizumab Drug: chemotherapy Radiation	Unknown status	Observational	/	Cancer Center, Union Hospital, Tongji Medical College, Huazhong University of Science and Technology, Wuhan, Hubei, China

Abbreviations: BMs, brain metastases; FSRT, fractionated stereotactic radiotherapy; HFSRT, hypofractionated stereotactic radiotherapy; ICIs, immune checkpoint inhibitors; IO, immunotherapy; NSCLC, non‐small‐cell lung cancer; RS, radiosurgery; RT, radiotherapy; SBRT, stereotactic body radiotherapy; SRS, stereotactic radiosurgery; SRT, stereotactic radiotherapy; WBRT, whole‐brain radiation therapy.

## AUTHOR CONTRIBUTIONS

Xiao‐Xia Zhu and Xiao‐Tong Duan carried out conceptualization. Zi‐Ying Chen and Xiao‐Tong Duan carried out article writing and editing. Xiao‐Xia Zhu and Si‐Miao Qiao carried out supervision. All authors read and approved the final manuscript. Zi‐Ying Chen and Xiao‐Tong Duan contributed equally to the manuscript.

## FUNDING INFORMATION

This work was supported by grants from the National Natural Science Foundation of China (81972853) and Clinical Research Startup Program of Southern Medical University by High‐level University Construction Funding of Guangdong Provincial Department of Education (LC2019ZD009).

## CONFLICT OF INTEREST

The authors declare that they have no conflict of interest.

## ETHICS AND CONSENT STATEMENT

Not applicable.

## Data Availability

Detailed data can be obtained by contacting the corresponding author.

## References

[1] Ray F , Ferlay J , Soerjomataram I , et al. Global cancer statistics 2018: GLOBOCAN estimates of incidence and mortality worldwide for 36 cancers in 185 countries. CA Cancer J Clin. 2018;68(6):394‐424. 30207593 10.3322/caac.21492

[2] Achrol AS , Rennert RC , Anders C , et al. Brain metastases. Nat Rev Dis Primers. 2019;5(1):1‐26. 30655533 10.1038/s41572-018-0055-y

[3] Akhoundova SD , Hüllner M , Kraft J , et al. P3.04‐22 response of brain metastases in patients with non‐small cell lung cancer treated with immunotherapy and brain directed radiotherapy. J Thorac Oncol. 2018;13(10):S930.

[4] Hubbeling HG , Schapira EF , Horick NK , et al. Safety of combined PD‐1 pathway inhibition and intracranial radiation therapy in non‐small cell lung cancer. J Thorac Oncol. 2018;3(4):550‐558. 10.1016/j.jtho.2018.01.012 29378267

[5] Schapira E , Hubbeling H , Shaw A , Oh KS , Gainor J , Shih HA . Local control and distant brain failure in patients treated with concurrent radiation and PD‐1/PDL‐1 inhibitors. Int J Radiat Biol. 2017;99(2):S210.

[6] McKelvey KJ , Hudson AL , Back M , et al. Radiation, inflammation and the immune response in cancer. Mamm Genome. 2018;29(11–12):843‐865. 30178305 10.1007/s00335-018-9777-0 PMC6267675

[7] Niesel K , Schulz M , Anthes J , et al. The immune suppressive microenvironment affects efficacy of radioimmunotherapy in brain metastasis. EMBO Mol Med. 2021;13(5):e13412. 33755340 10.15252/emmm.202013412 PMC8103101

[8] Li W , Yu H . Separating or combining immune checkpoint inhibitors (ICIs) and radiotherapy in the treatment of NSCLC brain metastases. J Cancer Res Clin Oncol. 2020;146(1):137‐152. 31813004 10.1007/s00432-019-03094-9 PMC11804339

[9] D'Souza NM , Fang P , Logan J , Yang J , Jiang W , Li J . Combining radiation therapy with immune checkpoint blockade for central nervous system malignancies. Front Oncol. 2016;6:212. 27774435 10.3389/fonc.2016.00212 PMC5053992

[10] Kroemer G , Galluzzi L , Kepp O , Zitvogel L . Immunogenic cell death in cancer therapy. Annu Rev Immunol. 2013;31:51‐72. 23157435 10.1146/annurev-immunol-032712-100008

[11] Kepp O , Senovilla L , Vitale I , et al. Consensus guidelines for the detection of immunogenic cell death. Onco Targets Ther. 2014;3(9):e955691. 10.4161/21624011.2014.955691 PMC4292729 25941621

[12] Jin Y , Kang Y , Peng XH , et al. Irradiation‐induced activated microglia affect brain metastatic colonization of NSCLC cells via miR‐9/CDH1 Axis. Onco Targets Ther. 2021;14:1911‐1922. 33758511 10.2147/OTT.S301412 PMC7981147

[13] Peng L , Wang Y , Fei S , et al. The effect of combining Endostar with radiotherapy on blood vessels, tumor‐associated macrophages, and T cells in brain metastases of Lewis lung cancer. Transl Lung Cancer Res. 2020;9(3):745‐760. 32676336 10.21037/tlcr-20-500 PMC7354151

[14] Vanpouille‐Box C , Alard A , Aryankalayil MJ , et al. DNA exonuclease Trex1 regulates radiotherapy‐induced tumour immunogenicity. Nat Commun. 2017;8:15618. 28598415 10.1038/ncomms15618 PMC5472757

[15] Hulsbergen AFC , Mammi M , Nagtegaal SHJ , et al. Programmed death receptor ligand one expression may independently predict survival in patients with non‐small cell lung carcinoma brain metastases receiving immunotherapy. Int J Radiat Oncol Biol Phys. 2020;108(1):258‐267. 32335185 10.1016/j.ijrobp.2020.04.018

[16] Takamori S , Toyokawa G , Takada K , Shoji F , Okamoto T , Maehara Y . Combination therapy of radiotherapy and anti‐PD‐1/PD‐L1 treatment in non‐small‐cell lung cancer: a mini‐review. Clin Lung Cancer. 2018;19(1):12‐16. 28739315 10.1016/j.cllc.2017.06.015

[17] Herter‐Sprie GS , Koyama S , Korideck H , et al. Synergy of radiotherapy and PD‐1 blockade in Kras‐mutant lung cancer. JCI Insight. 2016;1:e87415. 27699275 10.1172/jci.insight.87415 PMC5033933

[18] Dovedi SJ , Cheadle EJ , Popple AL , et al. Fractionated radiation therapy stimulates antitumor immunity mediated by both resident and infiltrating polyclonal T‐cell populations when combined with PD‐1 blockade. Clin Cancer Res. 2017;23(18):5514‐5526. 28533222 10.1158/1078-0432.CCR-16-1673

[19] Xia WY , Feng W , Zhang CC , et al. Radiotherapy for non‐small cell lung cancer in the immunotherapy era: the opportunity and challenge‐a narrative review. Transl Lung Cancer Res. 2020;9:2120‐2136. 33209631 10.21037/tlcr-20-827 PMC7653139

[20] Deng L , Liang H , Burnette B , et al. Irradiation and anti‐PD‐L1 treatment synergistically promote antitumor immunity in mice. Clin Invest. 2014;124:687‐695. 10.1172/JCI67313 PMC3904601 24382348

[21] Zeng J , See AP , Phallen J , et al. Anti‐PD‐1 blockade and stereotactic radiation produce long‐term survival in mice with intracranial gliomas. Int J Radiat Oncol Biol Phys. 2013;86:343‐349. 23462419 10.1016/j.ijrobp.2012.12.025 PMC3963403

[22] Lee WH , Sonntag WE , Lee YW . Aging attenuates radiation‐induced expression of pro‐inflammatory mediators in rat brain. Neurosci Lett. 2010;476:89‐93. 20385203 10.1016/j.neulet.2010.04.009 PMC2875775

[23] Twyman‐Saint Victor C , Rech AJ , Maity A , et al. Radiation and dual checkpoint blockade activate nonredundant immune mechanisms in cancer. Nature. 2015;520:373‐377. 25754329 10.1038/nature14292 PMC4401634

[24] Zhou S , Xie J , Huang Z , et al. Anti‐PD‐(L)1 immunotherapy for brain metastases in non‐small cell lung cancer: mechanisms, advances, and challenges. Cancer Lett. 2021;502:166‐179. 33450361 10.1016/j.canlet.2020.12.043

[25] Patruni S , Khattab A , Abel S , et al. A comparative analysis of survival in patients with non‐small cell lung cancer with brain metastases receiving intracranial radiation with and without immunotherapy. J Clin Oncol. 2019;37(15):9025 –9025.

[26] Shaverdian N , Lisberg AE , Bornazyan K , et al. Previous radiotherapy and the clinical activity and toxicity of pembrolizumab in the treatment of non‐small‐cell lung cancer: a secondary analysis of the KEYNOTE‐001 phase 1 trial. Lancet Oncol. 2017;18(7):895‐903. 28551359 10.1016/S1470-2045(17)30380-7 PMC5538772

[27] Ahmed KA , Kim S , Arrington J , et al. Outcomes targeting the PD‐1/PD‐L1 axis in conjunction with stereotactic radiation for patients with non‐small cell lung cancer brain metastases. J Neurooncol. 2017;133(2):331‐338. 28466250 10.1007/s11060-017-2437-5

[28] Chen L , Douglass J , Kleinberg L , et al. Concurrent immune checkpoint inhibitors and stereotactic radiosurgery for brain metastases in non‐small cell lung cancer, melanoma, and renal cell carcinoma. Int J Radiat Oncol Biol Phys. 2017;100(4):916‐925. 29485071 10.1016/j.ijrobp.2017.11.041

[29] Pike LRG , Bang A , Ott P , et al. Radiation and PD‐1 inhibition: favorable outcomes after brain‐directed radiation. Radiother Oncol. 2017;124(1):98‐103. 28662869 10.1016/j.radonc.2017.06.006

[30] Li J , Wang Y , Tang C , et al. Concurrent nivolumab and ipilimumab with brain stereotactic radiosurgery for brain metastases from non‐small cell lung cancer: a phase I trial. J Clin Oncol. 2020;38(15):2531 –2531.

[31] Porte J , Saint MC , Frederic MT , et al. Loco‐regional control and survival outcomes after combined stereotactic radiation therapy and immune checkpoint inhibitors for brain metastases from non‐small cell lung cancer. Int J Radiat Oncol Biol Phys. 2021;111(3):e577.

[32] Chen L , Diehl A , Yarchoan M , et al. Survival outcomes following combination radiotherapy and immune checkpoint inhibitors. Int J Radiat Oncol Biol Phys. 2017;99(2):E583.

[33] Chen L , Douglass J , Walker AJ , et al. Concurrent immunotherapy and stereotactic radiosurgery for brain metastases is associated with a decreased incidence of new intracranial metastases. Int J Radiat Oncol Biol Phys. 2015;93(3):E102.

[34] Srivastava A , Huang J . The impact of the timing of PD‐1 inhibition on disease control for brain metastases treated with stereotactic radiosurgery. Int J Radiat Oncol Biol Phys. 2017;99(2):E111.

[35] Dovedi SJ , Adlard AL , Lipowska‐Bhalla G , et al. Acquired resistance to fractionated radiotherapy can be overcome by concurrent PD‐L1 blockade. Cancer Res. 2014;74(19):5458‐5468. 25274032 10.1158/0008-5472.CAN-14-1258

[36] Imber BS , Hellmann MD , Kris MG , et al. Lesion response and intracranial control of brain metastases from nonsmall cell lung cancer after stereotactic radiosurgery or hypofractionated radiation therapy combined with checkpoint inhibitors. Int J Radiat Oncol Biol Phys. 2017;99(2):E465‐E466.

[37] Shepard MJ , Xu ZY , Donahue J , et al. Stereotactic radiosurgery with and without checkpoint inhibition for patients with metastatic non‐small cell lung cancer to the brain: a matched cohort study. J Neurosurg. 2019;undefined:1‐8. 10.3171/2019.4.JNS19822 31349225

[38] Levy A , Faivre‐Finn C , Hasan B , et al. Diversity of brain metastases screening and management in non‐small cell lung cancer in Europe: results of the European Organisation for Research and Treatment of Cancer Lung Cancer Group Survey. Eur J Cancer. 2018;93:37‐46. 29477100 10.1016/j.ejca.2018.01.067

[39] Gerber DE , Urbanic JJ , Langer C , et al. Treatment design and rationale for a randomized trial of cisplatin and etoposide plus thoracic radiotherapy followed by nivolumab or placebo for locally advanced non‐small‐cell lung cancer (RTOG 3505). Clin Lung Cancer. 2017;18(3):333‐339. 27923550 10.1016/j.cllc.2016.10.009 PMC5406261

[40] Iyengar P , Gerber DE . Locally advanced lung cancer: an optimal setting for vaccines and other immunotherapies. Cancer J. 2013;19(3):247‐262. 23708072 10.1097/PPO.0b013e318292e51a PMC3689291

[41] Schapira E , Hubbeling H , Yeap BY , et al. Improved overall survival and locoregional disease control with concurrent PD‐1 pathway inhibitors and stereotactic radiosurgery for lung cancer patients with brain metastases. Int J Radiat Oncol Biol Phys. 2018;101(3):624‐629. 29678530 10.1016/j.ijrobp.2018.02.175

[42] Martin AM , Cagney DN , Catalano PJ , et al. Immunotherapy and symptomatic radiation necrosis in patients with brain metastases treated with stereotactic radiation. JAMA Oncol. 2018;4(8):1123‐1124. 29327059 10.1001/jamaoncol.2017.3993 PMC5885198

[43] Singh C , Qian JM , Yu JB , Chiang VL . Local tumor response and survival outcomes after combined stereotactic radiosurgery and immunotherapy in non‐small cell lung cancer with brain metastases. J Neurosurg. 2019;132(2):512‐517. 30771783 10.3171/2018.10.JNS181371

[44] Matteucci P , Santo B , Ippolito E , et al. EP‐1231 immune checkpoint inhibitor and encephalic radiotherapy: toxicity and adverse events. Radiother Oncol. 2019;133:S678.

[45] Arscott WT , Zhu S , Plastaras JP , Maity A , Alonso‐Basanta M , Jones J . Acute neurologic toxicity of palliative radiotherapy for brain metastases in patients receiving immune checkpoint blockade. Neurooncol Pract. 2019;6(4):297‐304. 31386046 10.1093/nop/npy042 PMC6660815

[46] Lesueur P , Escande A , Thariat J , et al. Safety of combined PD‐1 pathway inhibition and radiation therapy for non‐small‐cell lung cancer: a multicentric retrospective study from the GFPC. Cancer Med. 2018;7(11):5505‐5513. 30311424 10.1002/cam4.1825 PMC6247050

[47] Kotecha R , Kim JM , Miller JA , et al. The impact of sequencing PD‐1/PD‐L1 inhibitors and stereotactic radiosurgery for patients with brain metastasis. Neuro Oncol. 2019;21(8):1060‐1068. 30796838 10.1093/neuonc/noz046 PMC6682202

[48] Akhoundova, D. , Hiltbrunner, S. , Mader, C. , et al. 18F‐FET PET for Diagnosis of Pseudoprogression of Brain Metastases in Patients With Non‐Small Cell Lung Cancer. Clin Nucl Med. 2019;45(2):113‐117. 10.1097/rlu.0000000000002890 31876831

[49] Jia, W. , Gao, Q. , Han, A. , et al. The potential mechanism, recognition and clinical significance of tumor pseudoprogression after immunotherapy. Cancer Bio & Med;16(4):655‐670. 10.20892/j.issn.2095-3941.2019.0144 PMC6936240 31908886

